# Addition of T2-guided optical tomography improves noncontrast breast magnetic resonance imaging diagnosis

**DOI:** 10.1186/s13058-017-0902-x

**Published:** 2017-10-24

**Authors:** Jinchao Feng, Junqing Xu, Shudong Jiang, Hong Yin, Yan Zhao, Jiang Gui, Ke Wang, Xiuhua Lv, Fang Ren, Brian W. Pogue, Keith D. Paulsen

**Affiliations:** 1 Thayer School of Engineering, Dartmouth College, 14 Engineering Drive, Hanover, NH 03755 USA; 2 Information Technology of Faculty, Beijing University of Technology, Beijing, 100124 China; 3 Department of Radiology, Xijing Hospital, Xi’an, 710032 China; 4 Geisel School of Medicine, Dartmouth College, Hanover, New Hampshire 03755 USA

**Keywords:** T2 MRI-guided, Near infrared spectral tomography, Breast cancer, Noncontrast MRI

## Abstract

**Background:**

While dynamic contrast-enhanced magnetic resonance imaging (DCE MRI) is recognized as the most sensitive examination for breast cancer detection, it has a substantial false positive rate and gadolinium (Gd) contrast agents are not universally well tolerated. As a result, alternatives to diagnosing breast cancer based on endogenous contrast are of growing interest. In this study, endogenous near-infrared spectral tomography (NIRST) guided by T2 MRI was evaluated to explore whether the combined imaging modality, which does not require contrast injection or involve ionizing radiation, can achieve acceptable diagnostic performance.

**Methods:**

Twenty-four subjects—16 with pathologically confirmed malignancy and 8 with benign abnormalities—were simultaneously imaged with MRI and NIRST prior to definitive pathological diagnosis. MRIs were evaluated independently by three breast radiologists blinded to the pathological results. Optical image reconstructions were constrained by grayscale values in the T2 MRI. MRI and NIRST images were used, alone and in combination, to estimate the diagnostic performance of the data. Outcomes were compared to DCE results.

**Results:**

Sensitivity, specificity, accuracy, and area under the curve (AUC) of noncontrast MRI when combined with T2-guided NIRST were 94%, 100%, 96%, and 0.95, respectively, whereas these values were 94%, 63%, 88%, and 0.81 for DCE MRI alone, and 88%, 88%, 88%, and 0.94 when DCE-guided NIRST was added.

**Conclusion:**

In this study, the overall accuracy of imaging diagnosis improved to 96% when T2-guided NIRST was added to noncontrast MRI alone, relative to 88% for DCE MRI, suggesting that similar or better diagnostic accuracy can be achieved without requiring a contrast agent.

**Electronic supplementary material:**

The online version of this article (doi:10.1186/s13058-017-0902-x) contains supplementary material, which is available to authorized users.

## Background

Clinically, magnetic resonance imaging (MRI) is recognized as the most sensitive examination for breast cancer surveillance [1–7]. However, dynamic contrast-enhanced (DCE) breast MRI has a substantial false positive rate [8, 9] due to its reliance on gadolinium (Gd) as a nonspecific contrast agent which produces high sensitivity, albeit with moderate specificity [10, 11]. Unfortunately, Gd-based contrast agents are not universally well tolerated because of the risk of adverse reactions [12] and contraindications in patients with impaired renal function [13]. The US Food and Drug Administration (FDA) has noted that higher than recommended doses or repeat doses of Gd appear to increase the risk for nephrogenic systemic fibrosis in those who have kidney disease [14]. The FDA is also investigating the risk of brain deposits with repeated use of Gd with MRI [15].

Relative to DCE MRI, T2 scans distinguish fibroglandular and vascular tissues from fat based on endogenous contrast achieved through loss in transverse magnetization due to spin dephasing from random interactions with surrounding molecules. Diffusion-weighted imaging (DWI) has been used to characterize breast lesions by measuring their random motion of free water protons with relatively high specificity (reported to be 84% in one meta-analysis) [10]. The combination of T2 and DWI has also been considered for diagnosis of breast cancer [16, 17]. Unfortunately, the diagnostic accuracy of either T2 or DWI alone or in combination has not been sufficient to replace DCE MRI in clinical breast imaging [16–19].

Near-infrared spectral tomography (NIRST) is attractive because it is noninvasive, fast, relatively inexpensive, and poses no risk of ionizing radiation [20–25]. In this case, NIRST illuminates the breast with multiple wavelengths of near-infrared light to image its optical properties from which hemoglobin concentration, oxygen saturation, water, and lipids, as well as scattering properties, can be inferred [20, 21, 26, 27]. While changes in these quantities (relative to their values in normal breast tissue) appear to be indicative of cancer, using them to detect and discriminate small breast abnormalities has not met clinical needs in diagnostic breast imaging to date.

Recovering the physiological properties accessible with NIRST by combining it with structural information available from another high (spatial) resolution imaging method may overcome this significant clinical limitation. Indeed, studies have shown that NIRST guided by MRI provides quantitative maps of optical properties [28–30], and may be a way of increasing the specificity of DCE MRI breast examinations [31]. The diagnostic advantages of combining optical image data with other breast imaging modalities have also been recognized [25, 27]. To date, only DCE MRI has been used to guide NIRST image reconstruction [31]. In this study, NIRST was guided by and added to noncontrast MRI to explore whether the combined image data, the acquisition of which does not require contrast injection or involve ionizing radiation, achieves acceptable diagnostic performance in a preliminary study of women with undiagnosed breast abnormalities at the time of the imaging examination.

## Methods

### Subjects

The imaging protocol for human subject participation was approved by the Committee for the Protection of Human Subjects at Dartmouth and Xijing Hospital in Xi’an, China. Women presenting with a clinical breast abnormality scheduled for surgical resection without known contraindications for MRI or Gd injection were invited to participate. Written informed consent was obtained from each participant. Twenty-four women were involved in the study and fell into an age range from 24 to 64 years. Of these 24 patients, 16 were found to have pathologically confirmed breast cancer and 8 had benign conditions. The average ages of the malignant and benign groups were 48 ± 11 years (range 24–64 years) and 31 ± 8 years (range 20–44 years), respectively. Mean body mass index (BMI) was 23.3 ± 3.6 kg/m^2^ while breast sizes were distributed as 10 A-cup, 8 B-cup, 3 C-cup, and 3 D-cup. Because most women in China do not participate in breast screening programs, patients did not have prior mammography. As a result, breast density was assessed based on MRI, and categorized as 1 fatty, 10 scattered, 7 heterogeneously dense, and 6 extremely dense. Subject data are summarized in Additional file 1 (Table S1).

### Imaging procedures

NIRST was performed with a hybrid frequency domain (FD)-continuous wave (CW) optical imaging system described in detail previously [30, 31]. The device included six FD wavelengths (spanning from 660 nm to 850 nm) and three CW wavelengths (900 nm to 950 nm). NIRST data were acquired at all nine wavelengths simultaneously with MRI by attaching an optical fiber interface to the commercial breast coil system already in place for clinical use [20]. To acquire the optical data, sixteen sequential source positions illuminated the breast through a custom optical switch. During each individual source illumination, the remaining 15 fibers detected transmitted light, yielding a total of 240 measurements at each wavelength. For FD measurement, the amplitude and phase of the detected light were collected by lock-in detection. For CW measurement, only amplitude data were recorded. The total NIRST imaging time per breast was 15 min.

MRI was performed using a Siemens MAGNETOM Trio 3.0 T scanner and involved standard clinical sequences. Specifically, acquisitions included a bilateral T1 precontrast scan (slice thickness < 3 mm), a unilateral T2 turbo spin echo sequence with fat suppression (slice thickness < 5 mm, TR/TE = 6490/61 ms, flip angle = 120°, voxel size = 1.0 × 1.0 × 4.0 mm, FOV = 332 mm^2^, and 100% FOV phase), bilateral DWI with eight b-values ranging from 0 to 1400 s/mm^2^, and a series of five bilateral T1 postcontrast scans spaced 90 s apart. Apparent diffusion coefficient (ADC) maps were calculated automatically by software on the MRI system. To achieve coregistration between MRI and NIRST data, fiducial markers were placed at the end of each NIRST fiber bundle.

### MRI image analysis

MRI examinations were evaluated independently by three radiologists experienced in diagnostic breast MRI (JX, more than 15 years; XL, more than 10 years; and KW, more than 8 years) who were blinded to pathological results; they scored the image data according to the Breast Imaging Reporting and Data System (BIRADS). Noncontrast images were assessed based on morphological features in the T2 scans with ADC values as references. In cases of disagreement, the final MR diagnosis (benign versus malignant) was based on majority consensus between the three radiologists. All lesions were measured along three orthogonal axes, and the greatest diameter was considered in the statistical analysis. In cases of multifocal or multicentric abnormalities, only the largest lesion was evaluated. DCE images were assessed according to Teifke criteria for contrast enhancement in focal breast lesions [32], which incorporates shape, border characteristics, enhancement kinetics, and enhancement pattern and maps to a BIRADS category based on cumulative scores [33]. Suspicious regions were manually segmented by a radiologist (JX) to create regions-of-interest (ROIs) for subsequent optical property assessment using either the T2 and DWI images (for noncontrast MRI analyses) or the DCE results (for comparative DCE MRI analyses). For DCE images, ROI segmentation was based on subtraction images formed by subtracting precontrast images from postcontrast images acquired 78 s after contrast injection. OsiriX image processing software (OsiriX MD 7.0, Pixmeo SARL, Bernex, Switzerland) was used to process all MRI data.

### NIRST image reconstruction guided by T2 MRI

**Fig. 1:**
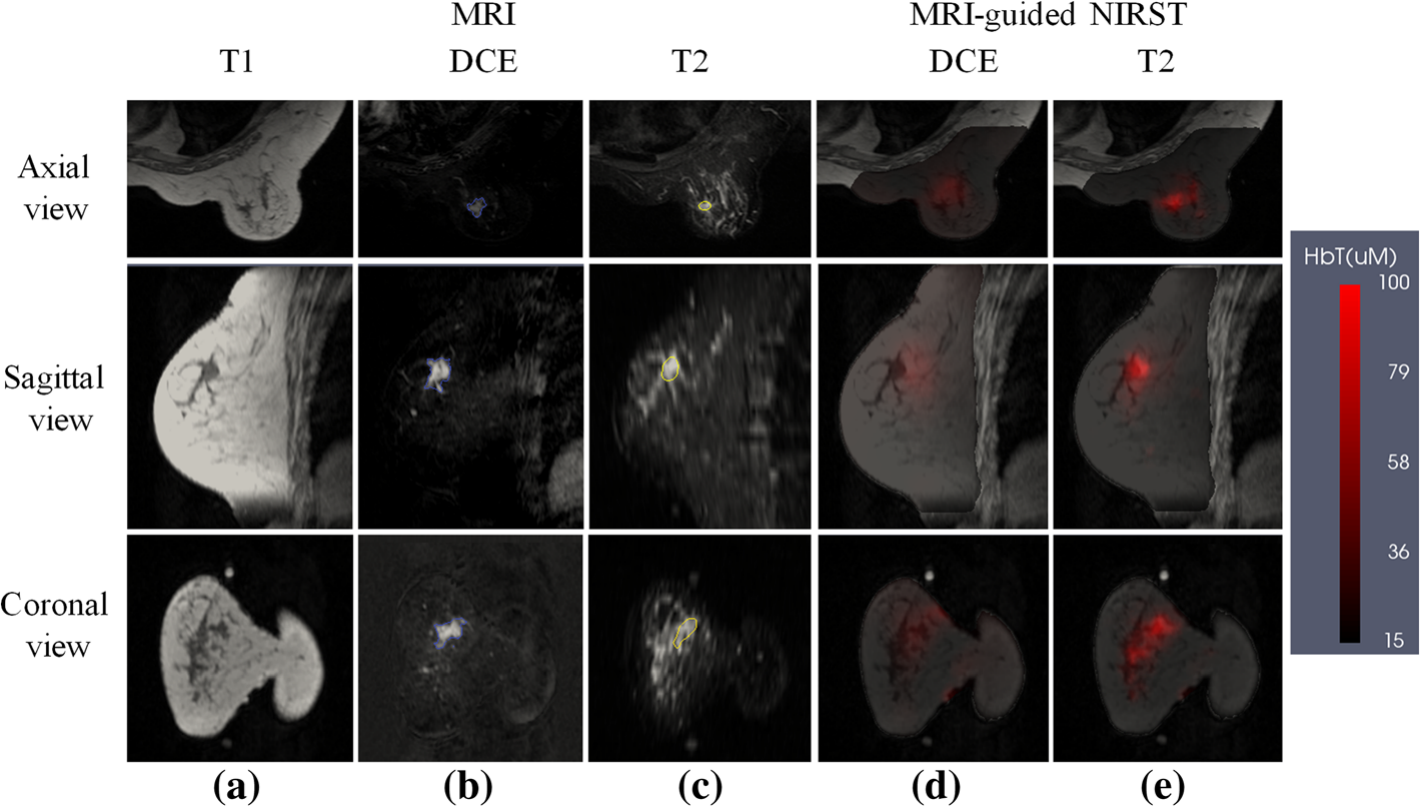
Example patient with a malignant lesion. **a** T1 MRI; **b** DCE MRI; **c** T2 MRI; **d**,**e** reconstructed HbT images with DCE-guided and T2-guided methods, respectively. Reconstructed images are overlaid on the T1 MRI cross-section. *DCE* dynamic contrast-enhanced, *HbT* total hemoglobin, *MRI* magnetic resonance imaging, *NIRST* near-infrared spectral tomography

Breast images were processed and reconstructed based on the open-source software platform NIRFAST [34]. Prior to NIRST reconstruction, a patient-specific finite element mesh was generated from T1 MR images (Fig. 1a). Then, data calibration was performed with a reference phantom to correct for small variations in detector response and light delivery, and to obtain initial estimates of optical properties for NIRST image reconstruction. Finally, NIRST image reconstruction was constrained by MR-derived spatial priors encoded through an automated direct regularization method that does not require MR segmentation [35], in which a weighted matrix, *L,* has the form:

$$ {L}_{ij}=\left\{\begin{array}{cc}\hfill 1\hfill & \hfill i=j\hfill \\ {}\hfill \hbox{-} \frac{1}{M_i}\exp \left(\hbox{-} \frac{{\left|{\gamma}_i\hbox{-} {\gamma}_j\right|}^2}{2{\sigma}_g^2}\right)\theta \left({\sigma}_d\hbox{-} \frac{\left|{r}_i\hbox{-} {r}_j\right|}{\max \left(\left|{r}_i\hbox{-} {r}_j\right|\right)}\right)\hfill & \hfill otherwise\hfill \end{array}\right. $$

where *γ*_*i*_ and *γ*_*j*_ are the grayscale values in the 16-bit MR image data (Fig. 1b for DCE-guided or Fig. 1c for T2-guided) mapped to nodes *i* and *j* on the FEM mesh, *r*_*i*_ and *r*_*j*_ are the coordinate positions of nodes *i* and *j. M*_*i*_ is a factor chosen for *i*th row in *L*, and satisfies ${M}_i=\sum_{j=1,j\ne i}^n{L}_{ij}\forall i=1,\dots, n$ where *n* is the number of finite element nodes. max(|*r*_*i*_ − *r*_*j*_|) is the maximum distance between any two finite element nodes. The function, *θ*(⋅), is the Heaviside step function, which determines the local weight applied to the *i*th NIRST image reconstruction position. *σ*_*g*_ is the characteristic grayscale difference over which to apply regularization, and *σ*_*d*_ is a factor related to the distance of influence of elements in the weight matrix relative to NIRST image position *i*. The operator, *L*, encodes optical property uniformity by penalizing similarly gray MR locations in the NIRST image to have similar update values at each iteration of the NIRST image reconstruction algorithm. In this study, parameters in *L* were fixed based on previous tests [35, 36], and set to be *σ*_*g*_ = 0.01, and *σ*_*d*_ = 0.4. The regularization parameters used in our experiments were 10 * max(*diag*(*J*_*k*_^*T*^ *J*_*k*_)), where *J*_*k*_ is the Jacobian matrix at the *k*th iteration in the update equation:

$$ \varDelta {x}_k={\left({J}_k^T{J}_k+\lambda {L}^TL\right)}^{-1}{J}_k^J\left(d-f\left({x}_{k-1}\right)\right) $$

where *Δx*_*k*_ is the update to the chromophore concentrations, *d* is the measured data, *f*(*x*_*k* − 1_) is the forward solution using the estimated parameters from the *k* − 1th iteration, and the superscript *T* denotes the transpose operation.

After NIRST image reconstruction, total hemoglobin (HbT) contrast was computed as the ratio of the average HbT in the abnormal ROI to the average HbT in the rest of the breast, and used to assess differences in benign and malignant abnormalities in subsequent diagnostic performance analyses. For evaluations based on noncontrast MRI data, ROIs segmented from the noncontrast MRI images were used whereas ROIs segmented from the DCE data were applied in the comparative DCE analysis.

### Statistical analysis

Student’s *t* tests were performed to access statistical differences in mean values (HbT contrast ratio, BIRADs score, and their combination) for breast abnormalities pathologically classified as benign or malignant. Receiver-operating characteristic (ROC) analysis was completed to evaluate differences in differentiation of benign versus malignant lesions as a function of cutoff value (HbT contrast ratio for NIRST, BIRADS score for MRI). The threshold corresponding to the largest summation of the average sensitivity and specificity on the ROC curve was considered to be the best cutoff point. To assess the combination of MRI and NIRST, multiple logistic regression coefficients were assembled into a single score which minimized the difference between the combined variables and the pathological diagnosis. Significance for all statistical tests was assumed at a confidence interval of 95% (*P* < 0.05) for a two-tailed distribution. The corresponding sensitivity and specificity in the ROC analysis are reported and NIRST reconstruction results guided by DCE MRI are also listed for comparison.

## Results

### Case 1: malignant finding

A woman with an undiagnosed 11 × 21 × 14 mm^3^ lesion in her right breast, later pathologically confirmed to be malignant, was imaged with MR-guided NIRST. Figure 1 shows NIRST HbT images overlaid on the corresponding T1 scans, based on DCE and T2 guidance. The DCE MRI result in Fig. 1b was formed by subtracting precontrast images from postcontrast images acquired 78 s after contrast injection. The sequence used to acquire the DCE MRI was nonfat-suppressed as shown in Fig. 1a. HbT contrast in the ROI (determined independently from DCE or noncontrast MRI image data) was 1.5 and 2.8, respectively, which is indicative of malignancy.

### Case 2: benign finding

**Fig. 2:**
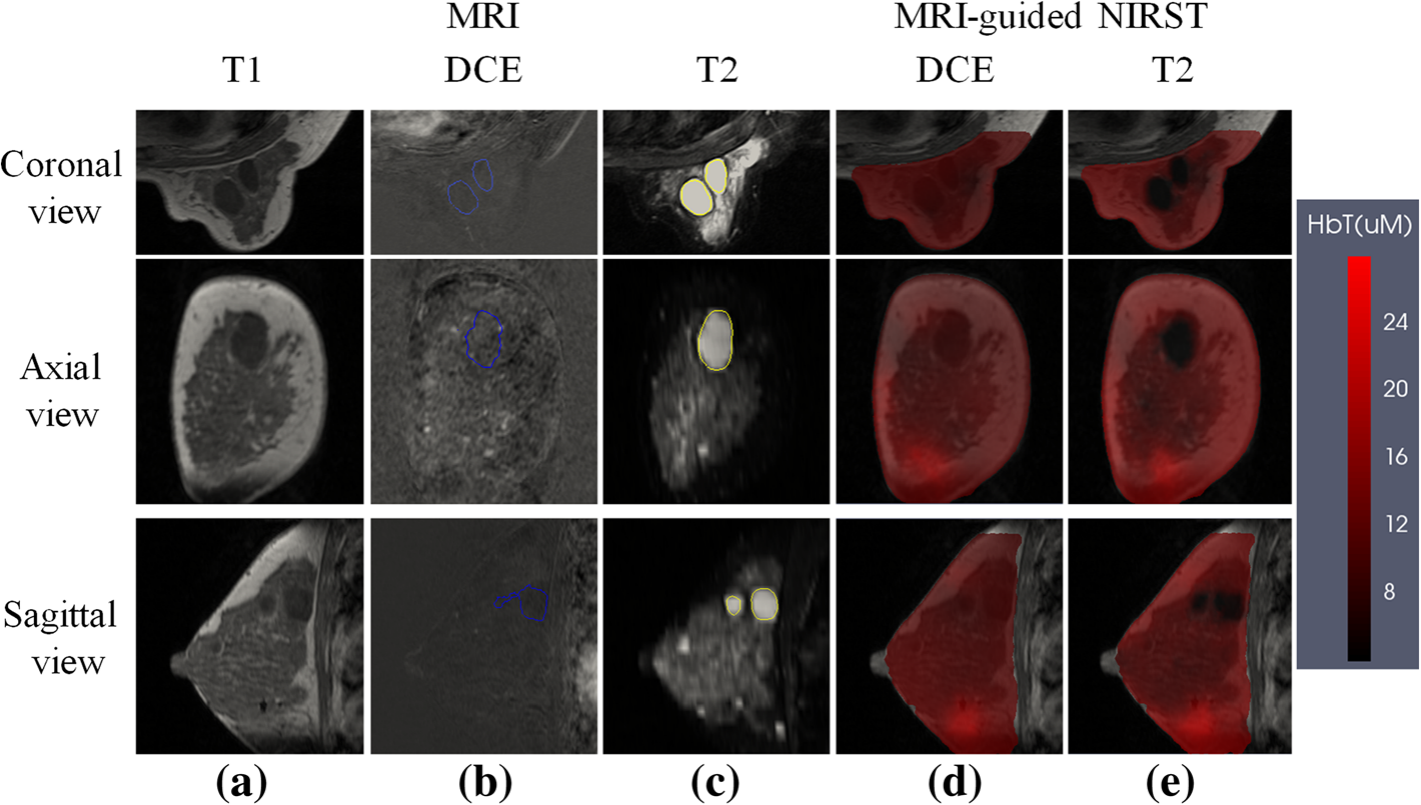
Example patient with a benign lesion. **a** T1 MRI; **b** DCE MRI; **c** T2 MRI; **d**,**e** Reconstructed HbT images with DCE-guided and T2-guided methods, respectively. Reconstructed images are overlaid on the T1 MRI cross-section. *DCE* dynamic contrast-enhanced, *HbT* total hemoglobin, *MRI* magnetic resonance imaging, *NIRST* near-infrared spectral tomography

A woman with a 21 × 34 × 26 mm^3^ abnormality in her left breast, pathologically confirmed as cystic hyperplasia, underwent a MR-guided NIRST examination prior to definitive diagnosis. Figure 2 shows representative MR and HbT images based on DCE- and T2-guided methods. As in Fig. 1, the DCE MRI acquisition was nonfat-suppressed, and postcontrast subtraction images are presented. HbT contrast in the ROI (determined independently from DCE or noncontrast MRI image data) was 0.9 and 0.5, respectively, for the two guidance methods, which is indicative of a benign lesion.

### Case 3: disagreement in diagnosis

**Fig. 3:**
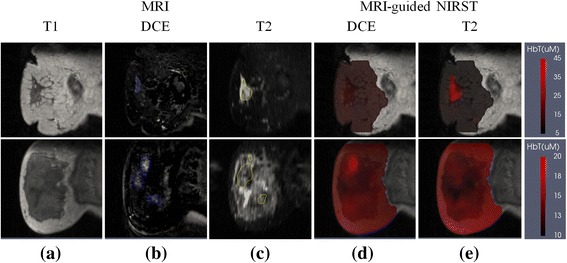
Two cases where the diagnosis produced with T2-guided NIRST disagreed with results derived from T2 + DWI or DCE MRI. *Top row*: a malignant case misdiagnosed by DCE MRI as a false negative. *Bottom row*: a benign case misdiagnosed by T2 + DWI or DCE MRI as a false positive. **a** T1 MRI; **b** DCE MRI; **c** T2 MRI; **d**,**e** Reconstructed HbT images with DCE-guided and T2-guided methods, respectively. Reconstructed images are overlaid on the T1 MRI cross-section. *DCE* dynamic contrast-enhanced, *HbT* total hemoglobin, *MRI* magnetic resonance imaging, *NIRST* near-infrared spectral tomography

Figure 3 presents two cases where noncontrast MRI and/or DCE MRI images resulted in misdiagnoses. The top row contains a false negative case in which the subject had a 10 × 25 × 25 mm^3^ lesion in her right breast that was interpreted as benign based on T2 + DWI MRI. Data from NIRST indicated the abnormality was malignant given its HbT contrast (average HbT in abnormal ROI to the average HbT in the rest of the breast) of 3.3 (higher than the cutoff value of 1.1) extracted from the T2-guided NIRST images. Postsurgical pathological results confirmed the tissue was malignant.

The bottom row of Fig. 3 shows a noncontrast MRI and DCE MRI false positive case where the diagnosis based on either noncontrast MRI or DCE MRI was malignant. In comparison, the T2 MRI did not exhibit much regional enhancement, and was used to guide HbT contrast from NIRST to reveal a value of 0.9 (lower than the cutoff value of 1.1) suggesting a benign diagnosis. Postsurgical pathological analysis confirmed the tissue was benign (adenosis).

### Diagnostic performance

Diagnostic performances of MRI alone, MR-guided NIRST alone, and the combination of MRI with NIRST are summarized in Table 1. Assuming a BIRADS 4 (or higher) image rating to be positive for cancer, noncontrast-enhanced MRI (T2 + DWI) yielded sensitivity, specificity, and diagnostic accuracy of 88%, 88%, and 88%, respectively, compared to values of 100%, 63%, and 88% for DCE MRI using the same cut-off (i.e., BIRADS ≥ 4 as a cancer diagnosis). ROC analysis of MRI alone yielded area under the curve (AUC) of 0.88 and 0.81 in the noncontrast MRI and DCE MRI cases. Standard deviations in the sensitivity, specificity, and accuracy results from the MRI assessments performed independently by the three radiologists were 6%, 0%, and 4%, respectively.

**Table 1:** Diagnostic performance of MRI, MR-guided NIRST, and MRI combined with NIRST

	MRI		MRI-guided NIRST		Combined MRI and NIRST	
	DCE	T2 + DWI	DCE-guided	T2-guided	MRI DCE + DCE-guided	MRI T2 + DWI + T2-guided
*P* values	<0.001	<0.001	0.001	<0.001	0.001	0.002
AUC	0.81	0.88	0.90	0.91	0.94	0.95
Sensitivity	0.94	0.88	0.88	0.88	0.88	0.94
Specificity	0.63	0.88	0.88	0.88	0.88	1
Accuracy	0.88	0.88	0.88	0.88	0.88	0.96

*n* = 24 patients

*P* values result from statistical tests of the mean diagnostic parameter in benign versus malignant cases classified by pathology

*AUC* area under the curve, *DCE* dynamic contrast-enhanced, *MRI* magnetic resonance imaging, *NIRST* near-infrared spectral tomography

**Fig. 4:**
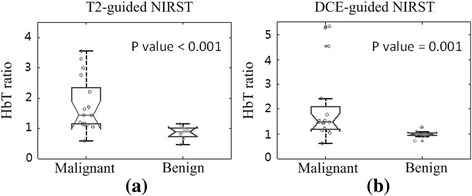
Boxplots of HbT contrast in malignant (*n* = 16) and benign (*n* = 8) groups obtained by **a** T2-guided and **b** DCE-guided methods, respectively. *DCE* dynamic contrast-enhanced, *HbT* total hemoglobin, *NIRST* near-infrared spectral tomography

**Fig. 5:**
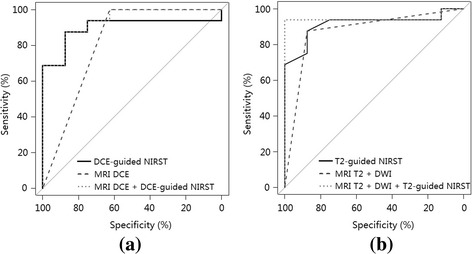
ROC curves for **a** DCE-guided NIRST, DCE MRI, and combined DCE MRI and DCE-guided NIRST, and **b** T2-guided NIRST, T2 + DWI MRI, and combined T2 + DWI MRI and T2-guided NIRST. *DCE* dynamic contrast-enhanced, *MRI* magnetic resonance imaging, *NIRST* near-infrared spectral tomography

For T2- and DCE-guided NIRST, a significant difference was found in mean HbT contrast (*P* ≤ 0.001) between pathologically confirmed malignant and benign abnormalities in the study participants (Fig. 4). For an HbT contrast cutoff value of 1.1, sensitivity, specificity, and diagnostic accuracy were 88% for both T2- and DCE-guided NIRST breast examinations. ROC analysis (Fig. 5) yielded AUCs of 0.91 and 0.90 in the two guidance cases.

When noncontrast MRI (T2 + DWI) and T2-guided NIRST were combined, sensitivity, specificity, diagnostic accuracy, and AUC increased to 94%, 100%, 96%, and 0.95, respectively. When DCE MRI was combined with DCE-guided NIRST, sensitivity, specificity, and accuracy remained at 88%, although AUC increased to 0.94.

No statistically significant difference was found in the diagnostic performance of noncontrast MRI relative to DCE MRI (*P* = 0.6), or from the combination of noncontrast-enhanced MRI (T2 + DWI) and T2-guided NIRST relative to the combination of DCE MRI and DCE-guided NIRST (*P* = 0.89).

## Discussion

While DCE is the clinically accepted standard for breast MRI, it has two important limitations: moderate specificity (~80%) and Gd contrast injection that increases examination time and cost, and risk of side effects [37, 38]. As a result, interest in T2 and DWI acquisitions has continued [16–19]. For example, 78% sensitivity and 87% specificity for breast cancer detection was achieved with noncontrast MRI in a recent study of 67 women [11]. However, the diagnostic performance of T2 sequences, with or without DWI, has not been sufficient to replace DCE MRI in clinical breast imaging to date.

To the best of our knowledge, this study is the first to investigate combinations of MR-guided NIRST based on T2 and DWI sequences to differentiate benign from malignant breast abnormalities. The approach yielded a sensitivity of 94%, specificity of 100% and diagnostic accuracy of 96%, which was improved over the performance achieved with T2 and DWI MRI alone and better than that attained with DCE MRI and DCE MR-guided NIRST [31], although statistically significant differences were not demonstrated. The importance of this finding is that MRI when combined with NIRST may achieve clinically acceptable diagnostic performance in women without contrast injection.

Other noncontrast-injection breast imaging methods, including x-rays [27] and ultrasound (US) [25], have been combined with optical tomography. Fang et al. [27] found statistically significant differences in optical properties of pathologically confirmed benign versus malignant breast abnormalities. Contrast in absolute and normalized HbT in cancers was 1.4 or less on average, or 1.2 or less in benign conditions. No estimates of diagnostic performance based on the data were reported. Statistically significant differences in optical properties were also presented by Zhu et al. [25] when optical imaging was guided by US, and estimates of sensitivity and specificity ranged from 97% to 100%, and 77% to 83%, respectively, for two reading radiologists when US and optical data were combined and imaging results were compared to pathology classifications. Relative to these reports, the combination of noncontrast MRI and T2-guided NIRST offered less sensitivity (94%) but better specificity (100%).

The sample size in this study is small, and is an important limitation in extrapolating results to larger numbers of patients. However, our focus in this study was to demonstrate that the diagnostic performance of noncontrast MRI for breast cancer detection could be improved by adding NIRST during an imaging trial. While the results of this limited patient cohort showed that the specificity of T2 + DWI MRI was better than that of DCE MRI, the trial was not designed to answer a mechanistic physiological hypothesis. Our interpretation is that the NIRST guided by T2/DWI or DCE would be similar in a larger cohort but that, in general, T2/DWI weighted by water is sufficiently similar to DCE to provide the guidance needed for NIRST recovery. Clearly lesions which have DCE contrast will also have some level of T2/DWI contrast. It is unlikely that this will be true for all lesions, but larger patient studies would be needed to tease out which lesions are more readily imaged by T2/DWI NIRST versus DCE NIRST. Additionally, the breast abnormalities evaluated were presented clinically prior to imaging, and do not reflect the full population expected in diagnostic breast MRI. Nonetheless, the study is the first to examine how a noncontrast MRI breast examination, when augmented by simultaneous optical imaging, compares to DCE MRI, and even DCE MRI when combined with NIRST in the same group of patients with undiagnosed breast abnormalities at the time of the imaging examination. Results showed that the diagnostic accuracy of combining T2-guided NIRST with noncontrast MRI was 96%, which was better than the performance of DCE MRI (88%) in the subjects evaluated. Enrollment of women in a larger study will allow more definitive estimates of the diagnostic performance of endogenous contrast in breast abnormalities examined with MRI combined with NIRST.

Interestingly, subjects with breast abnormalities that proved to be benign were predominantly premenopausal (7/8) and had denser breast tissue. While this study did not contribute confirmatory data, the diagnosis of benign lesions in postmenopausal subjects with less dense breasts would likely be improved compared to the results shown here. Indeed, the quality of T2 + DWI and NIRST images in less dense breast tissue is typically better than in the dense breast, in part because the signal to noise ratio of detected optical signals is usually much higher. Thus, we would expect that the T2 + DWI and NIRST diagnostic performance would improve in classifying benign lesions in postmenopausal breasts that are more likely to have lower radiographic densities.

In addition to using HbT contrast to assess differences in the benign and malignant breast abnormalities that were imaged, other optical chromophores such as oxygen saturation, water, lipids, and scattering properties were also extracted from the images of all patients. A statistically significant difference between the benign and cancer cases was found in HbT contrast but not in any of these other parameters individually, and we did not evaluate multiparameter indices or consider combinations of properties in the analyses reported here.

The MRI-guided NIRST reconstruction method used in this study is influenced by the MRI grayscale contrast and, as a result, both the ROI volume and the regularization scaling are different depending on whether T2 MRI or DCE MRI is applied. These differences led to the differences observed in the recovered HbT contrast obtained with the two methods in individual subjects. Information on grayscale contrast and ROI volume has been added to Additional file 1 (Table S1) so that these differences can be appreciated in each case.

ROIs were defined based on the morphology but not the brightness of the T2 signal. Although the T2 signal of many breast lesions, especially malignant lesions, is brighter than the surrounding normal tissue, the degree of enhancement depends on the specifics of pathological tissue composition and/or lesion progression (i.e., necrotic fibrosis in lesions, and so forth); hence, the T2 signal in some lesions is darker than the surrounding normal tissue, as in Fig. 3c. Moreover, the T2 signal levels in some lesions are heterogeneously mixed, being brighter and darker in neighboring areas. Figure 3c is a good example of the difficulty in identifying breast cancers without specific radiology training and experience, and how optical imaging can add diagnostic power to noncontrast breast MRI.

## Conclusions

In this study, women with undiagnosed breast abnormalities were imaged with noncontrast MRI combined with NIRST. The overall accuracy of imaging diagnosis improved to 96% when T2-guided NIRST was added to noncontrast MRI alone, relative to 88% for DCE MRI. This result suggests that T2 MRI-guided NIRST may offer diagnostic imaging of breast abnormalities with accuracy comparable to DCE MRI in patients for whom contrast examinations are contraindicated.

## Additional file

Additional file 1: Table S1. Complete MRI and optical data from 24 patients analyzed. Data in the DCE and T2 + DWI columns are the results of radiologist interpretation, where “1” and “0” indicate malignant and benign diagnoses, respectively. Data in DCE-NIRST and T2-NIRST columns are the contrast ratio of HbT in the abnormal ROI relative to the rest of the breast. (PDF 32 kb)

## Abbreviations

ADC: Apparent diffusion coefficient
AUC: Area under the curve
BIRADS: Breast Imaging Reporting and Data System
CW: Continuous wave
DCE: Dynamic contrast-enhanced
DWI: Diffusion-weighted imaging
FD: Frequency domain
FDA: Food and Drug Administration
Gd: Gadolinium
HbT: Total hemoglobin
MRI: Magnetic resonance imaging
NIRST: Near-infrared spectral tomography
ROC: Receiver-operating characteristic
ROI: Regions of interest
US: Ultrasound
