# Supplementary material for: Addition of T2-guided optical tomography improves noncontrast breast magnetic resonance imaging diagnosis
Source: Breast Cancer Res. 2017 Oct 24;19:117. doi: 10.1186/s13058-017-0902-x (PMC5655871; doi:10.1186/s13058-017-0902-x)
Supplement: Additional file 1: Table S1. — Complete MRI and optical data from 24 patients analyzed. Data in the DCE and T2 + DWI columns are the results of radiologist interpretation, where “1” and “0” indicate malignant and benign diagnoses, respectively. Data in DCE-NIRST and T2-NIRST columns are the contrast ratio of HbT in the abnormal ROI relative to the rest of the breast. (PDF 32 kb) [file 13058_2017_902_MOESM1_ESM.pdf]

Pat. ID	Pathology	Breast Density	Tumor Size (mm^3)	ROI Volume (mm^3)		Gray Scale Contrast		DCE			T2+DWI			DCE-NIRST	T2-NIRST
				DCE	T2	DCE	T2	R1	R2	R3	R1	R2	R3		
1	IDC	HD	25×22×**26**	4357	3253	5.3	4.9	0	1	1	1	1	1	2.4	1.1
2	IDC	S	10×**25**×**25**	7412	4744	5.0	4.5	1	1	1	0	0	0	0.6	0.6
3	IDC	HD	9×**16**×**16**	1626	4975	5.0	2.9	1	1	1	1	1	1	1.0	1.2
4	IDC	HD	11×**16**×14	1297	2553	18.9	2.5	1	1	1	1	1	1	5.3	1.7
5	IDC	S	11×17×**23**	2082	2441	7.7	4.4	1	1	1	1	1	1	1.5	1.6
6	IDC	ED	23×40×**70**	16304	17352	7.1	3.5	1	1	1	1	1	1	1.5	1.1
7	IDC	S	12×**13**×8	5425	4403	1.6	2.7	1	1	0	1	1	0	1.1	1.2
8	IDC	S	35×46×**74**	20452	16134	7.0	2.9	1	1	1	0	1	1	1.5	1.1
9	IDC	S	10×**25**×**25**	1662	1028	3.0	5.6	1	1	1	0	0	0	1.1	3.3
10	DC	HD	21×18×**25**	4846	4740	7.2	4.8	1	1	1	1	1	1	1.2	1.1
11	IDC	S	11×**21**×14	3459	1717	0.5	3.9	1	1	1	1	1	1	1.5	2.8
12	IDC	S	15×25×**42**	9477	6871	7.7	4.1	1	1	1	0	1	1	1.77	2.2
13	IDC	S	22×20×**32**	8936	4910	9.1	4.6	1	1	1	1	1	1	1.6	1.4
14	IDC	HD	20×**59**×40	20574	14227	10.9	3.8	1	1	1	1	1	1	1.4	1.4
15	IDC	S	15×20×**29**	2701	2899	8.8	4.5	1	1	1	1	1	1	5.3	1.7
16	IDC	F	20×27×**33**	7102	8989	16.6	3.5	1	1	1	1	1	1	4.5	3.0
17	FA	ED	10×**20**×19	6340	2591	2.1	2.0	1	0	0	0	0	0	1.0	0.7

18	AD	ED	RD	22154	14265	3.4	1.7	0	1	1	1	1	0	1.1	0.9
19	FA	HD	29×33×**58**	18344	13901	1.6	0.8	0	0	0	0	0	0	1.0	1.0
20	FA	ED	12×20×**21**	575	1292	3.6	2.3	0	0	0	0	0	0	0.7	0.8
21	IP	HD	6×7×**9**	1160	1447	3.4	2.6	1	1	1	0	0	0	1.3	1.1
22	AD	S	15×18×**26**	3646	2370	0.4	4.5	0	1	1	0	0	1	1.0	1.1
23	FA	ED	**27**×25×17	4861	4291	6.4	3.9	0	0	0	0	0	0	1.0	0.9
24	CH	ED	21×**34**×26	13646	13668	0.9	4.7	0	0	0	0	0	0	0.9	0.5

Pathological result: IDC: Invasive Ductal Carcinoma; FA: Fibroadenoma; AD: Adenosis;
IP: Intraductal Papilloma; CH: Cystic Hyperplasia;
Breast density: S: Scattered; ED: Extremely Dense; HD: Heterogeneously Dense; F: Fatty;
Size: RD: Regional Distribution;
Radiologists: R1: Dr. Xu; R2: Dr. Lu; R3: Dr. Wang
